# The association between temporal changes in the use of obstetrical intervention and small- for-gestational age live births

**DOI:** 10.1186/s12884-015-0670-5

**Published:** 2015-09-29

**Authors:** Amy Metcalfe, Sarka Lisonkova, KS Joseph

**Affiliations:** Department of Obstetrics and Gynecology, University of Calgary, Calgary, Canada; Department of Obstetrics and Gynecology, University of British Columbia and the Children’s and Women’s Hospital and Health Centre of British Columbia, Vancouver, Canada; School of Population and Public Health, University of British Columbia, Vancouver, Canada

**Keywords:** Small for gestational age, Labour induction, Cesarean section, Preterm birth, Time trends

## Abstract

**Background:**

The literature attributes secular declines in small-for-gestational age (SGA) live births to changes in maternal smoking and other maternal characteristics. However, there are reasons to believe that the observed reductions in SGA may be a consequence of early delivery following obstetric intervention.

**Methods:**

We examined temporal trends in obstetrical intervention and SGA among singleton live births in the United States from 1990 to 2010. The modified Kitagawa decomposition, based on the fetuses-at-risk approach, was used to assess the relative contribution of changes in the gestational age distribution and gestational age-specific SGA to overall changes in SGA. Reductions in SGA rates due to a left shift in the gestational age distribution were assumed to primarily reflect increased obstetrical intervention, whereas decreases in overall SGA due to decreases in gestational-age-specific SGA rates were assumed to reflect declines in risk factors.

**Results:**

Temporal trends in SGA followed a non-linear pattern, with substantial declines from 10.1 % in 1990–92 to 8.9 % in 2002–04, followed by a small increase to 9.1 % in 2008–10. Rates of maternal smoking steadily decreased throughout the same time period and changes in SGA rates were more consistent with changes in the gestational age distribution. The modified Kitagawa decomposition analysis also attributed the initial decline in SGA rates to changes in the gestational age distribution.

**Conclusions:**

Complex temporal pattern in SGA rates cannot be explained by the linear pattern of changes in factors like maternal smoking. Changes in the gestational age distribution are more consistent with the observed secular trends in SGA rates.

## Background

Iatrogenic early delivery through labour induction or cesarean delivery given suspected fetal or maternal compromise is part of the foundation of modern obstetrics [1]. Increasing rates of obstetric intervention in industrialized countries have led to a shift in the population distribution of gestational age at delivery, with increases in the rates of preterm and early term birth and declines in the rates of post-term birth (1). Labour induction rates in the United States (US) among term (≥37 weeks) singleton deliveries increased from 10.9 % in 1991 to 24.0 % in 2006 [2]. Similarly, 15.6 % of preterm (<37 weeks) singleton deliveries in the US had labour induced in 2006-an increase of 105 % from 1991 [2]. The proportion of singleton infants delivered at term via cesarean section also increased over this period from 21.4 % in 1991 to 28.7 % in 2006, while cesarean rates among preterm infants increased from 25.1 % in 1991 to 36.9 % in 2006 [2]. The largest increase in the rate of obstetrical intervention among preterm deliveries was observed at late preterm gestation (34–36 weeks) [2].

The fetuses-at-risk model, which shows that small-for-gestational age (SGA) births increase with increasing gestational duration [1], and empirical evidence [3, 4] suggest that increasing rates of labour induction and cesarean delivery without labour can lead to decreases in SGA births. Delivery at an earlier gestation could result in an appropriately grown fetus rather than one that would have delivered spontaneously at later gestation as an SGA infant after a period of impaired growth. Perhaps the most compelling evidence for this proposition comes from the Disproportionate Intrauterine Growth Intervention Trial at Term (DIGITAT) trial [5] which randomized women suspected of having a growth-restricted fetus at near term or term gestation to labour induction or expectant management. The labour induction group was delivered 10 days earlier on average and had a mean birthweight that was 130 g lower than the expectant management group [5]. Nevertheless, rates of SGA (<3rd percentile) were substantially lower in the labour induction group (12.5 %) compared with the expectant management group (30.6 %) [5]. On the other hand, non-experimental studies examining temporal trends in SGA, and ecological studies on SGA and obstetrical intervention have yielded conflicting results [6–8]. Nevertheless, the prevalent opinion in the current literature appears to be that changes in maternal characteristics (such as the decline in maternal smoking and the increase in maternal pre-pregnancy body mass index) were responsible for the observed temporal improvements in SGA rates [6, 9]. We therefore carried out a study to clarify the role of obstetrical intervention (including labour induction and cesarean delivery) on temporal declines in SGA rates by examining the relative contribution of the shift in the gestational age distribution and changes in the gestational-age-specific SGA rates to changes in overall SGA. Reductions in overall SGA rates due to a left shift in the gestational age distribution were assumed to primarily reflect increased obstetrical intervention, whereas decreases in overall SGA due to decreases in gestational-age-specific SGA rates were assumed to reflect declines in risk factors such as maternal smoking.

## Methods

Population-based data on singleton live births in the US from 1990 to 2010 were obtained from the National Centre for Health Statistics (NCHS). These NCHS data were abstracted from birth certificates and included information on patient demographics, medical interventions during delivery, and birth outcomes. NCHS data is publicly available for research purposes (http://www.cdc.gov/nchs/). Ethics approval for this study was granted by the Research Ethics Board at the University of British Columbia. The study was limited to singleton live births delivered between 24 and 43 weeks of gestation, with gestational age based on the clinical estimate. Infants with a birthweight <500 g and <24 weeks of gestation were excluded to minimize biases associated with variable practices in registering births at the edge of viability. Infants were also excluded if they were missing data on birthweight, clinical estimate of gestational age, sex, labour induction, mode of delivery or had a congenital anomaly documented on the birth certificate. A total of 71,678,794 infants were available for analysis. Small-for-gestational age live births were defined as those with a birthweight <10th percentile for gestational age using the United States population reference [10]. These neonatal growth curves were based on US births in 1991 [10].

Our analysis used the fetuses-at-risk approach [1, 11] to calculate temporal trends in the gestational-age specific rates of SGA and obstetrical intervention. This longitudinal perspective treats gestational age as survival time and can provide causal insights into obstetric intervention and its impact on fetal growth restriction. It was preferred to the traditional formulation of gestational age-specific rate of SGA because the latter represents a cross sectional view of the frequency of obstetric intervention and SGA at each gestational week [1, 11]. Gestational age-specific SGA rates were calculated by dividing the number of SGA live births at any gestational age by the number of fetuses at risk of being born SGA at that gestational age. Gestational age-specific rates of SGA and obstetrical intervention were examined within 3 year periods to ensure stable estimates. Rate ratios (with 95 % confidence intervals) and the Cochrane-Armitage test for trend were used to assess differences over time.

The modified Kitagawa decomposition based on the fetuses-at-risk framework [12, 13] was used to assess the impact of temporal changes in the gestational age distribution and temporal changes in the gestational age-specific SGA rate on overall temporal changes in the rate of SGA. The Kitagawa decomposition formula [12, 13] is given below:

$$ N1-N2 = {\displaystyle \sum_{i=1}^n}\frac{\left({R}_{1i} + {R}_{2i}\right)}{2}\ \left({F}_{1i} - {F}_{2i}\right) + {\displaystyle \sum_{i=1}^n}\frac{\left({F}_{1i} + {F}_{2i}\right)}{2}\left({R}_{1i} - {R}_{2i}\right) $$

N refers to the rate of SGA in periods 1 and 2; R represents the rate of SGA for a given gestational age (*i*); while F indicates the proportion of fetuses at risk for a given gestational age (*i*). The first part of this equation quantifies the impact of temporal changes in the gestational age distribution on temporal changes in SGA rates, while the second part of the equation quantifies the impact of temporal changes in gestational age-specific SGA rates on temporal changes in SGA rates. These analyses were carried out among all live births and repeated among live births to smokers (since reductions in maternal smoking are thought to be a key factor in the observed decline in SGA rates). We hypothesized that the results of the modified Kitagawa decomposition would differ between all live births and live births to smokers if temporal reductions in smoking had in fact contributed substantially to temporal changes in SGA rates. The Kitagawa analysis was favoured over regression because disentangling the contribution of risk factors (such as smoking) and obstetrical interventions (such as labour induction) on SGA can be challenging due to confounding by indication [14, 15]. All analyses were conducted using Stata SE Version 12.

## Results

**Fig. 1:**
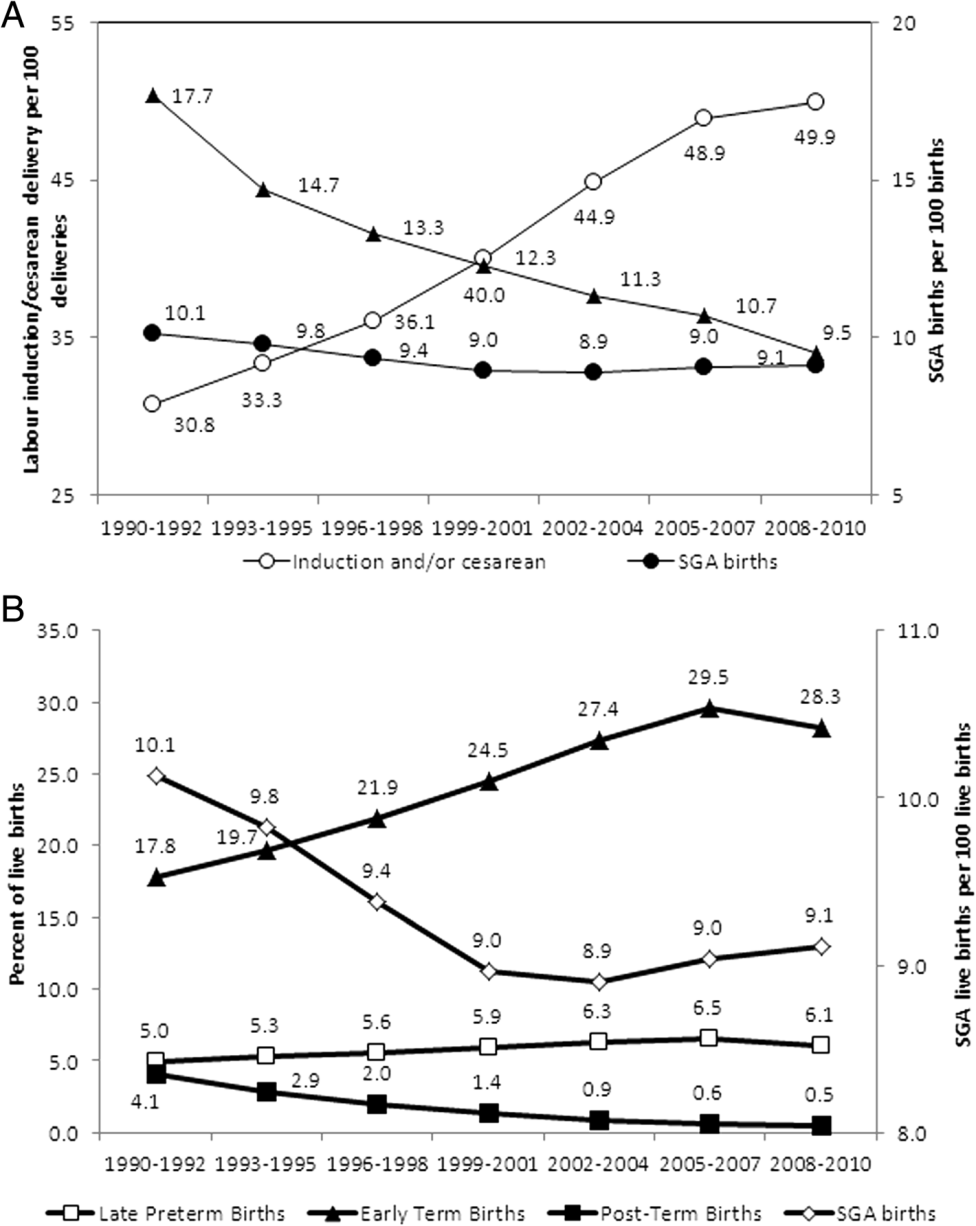
Temporal trends in rates of labour induction and/or cesarean delivery, maternal smoking and small-for-gestational age (SGA) births (**a**) and temporal trends in rates of late preterm, early term, post-term and SGA births (**b**) among singleton live births in the United States, 1990 to 2010

The rate of SGA steadily decreased from 10.1 % (95 % CI 10.1–10.2) in 1990–92 to 8.9 % (95 % CI 8.9–8.9) in 2002–04 (rate ratio 0.88, 95 % CI 0.88–0.88) against a background of increasing rates of obstetrical intervention. Rates of labour induction and/or cesarean delivery rose from 30.8 % (95 % CI 30.7–30.8) in 1990–92 to 44.9 % (95 % CI 44.8–44.9) in 2002–04 (46 % increase, *p* < 0.001, Fig. 1a). However, a different pattern in SGA and obstetric intervention rates was observed between 2002–04 and 2008–10. While rates of obstetrical intervention increased slightly between 2002–04 and 2008–10, with 49.9 % (95 % CI 49.9–50.0) of deliveries in 2008–10 involving labour induction, cesarean delivery or both (11 % increase, *p* < 0.001), rates of SGA increased slightly, but significantly, from 8.9 % in 2002–04 to 9.1 % (95 % CI: 9.1–9.1) in 2008–2010 (Fig. 1). Rates of maternal smoking declined steadily throughout this time period from 17.7 % (95 % CI 17.7–17.8) in 1990–92 to 9.5 % (95 % CI: 9.5–9.5) in 2008–10 (Fig. 1a). Obstetrical intervention rates among smokers mimicked those among all women, with rates of 30.2 % (95 % CI: 30.1–30.3) in 1990–92, 46.2 % (95 % CI: 46.1–46.3) in 2002–04, and 53.5 % (95 % CI: 53.4–53.6) in 2008–10.

The increases in obstetrical intervention were accompanied by changes in the gestational age distribution (Fig. 1b). Rates of late preterm birth increased from 5.0 % in 1990–92 to 6.5 % in 2005–07 before declining to 6.1 % in 2008–10, while rates of early term birth increased from 17.8 % in 1990–92 to 29.5 % in 2005–07 before declining to 28.3 % in 2008–10. Rates of post-term birth (≥42 weeks) decreased from 4.1 % in 1990–92 to 0.6 % in 2005–07 before leveling off at 0.5 % in 2008–10.

**Fig. 2:**
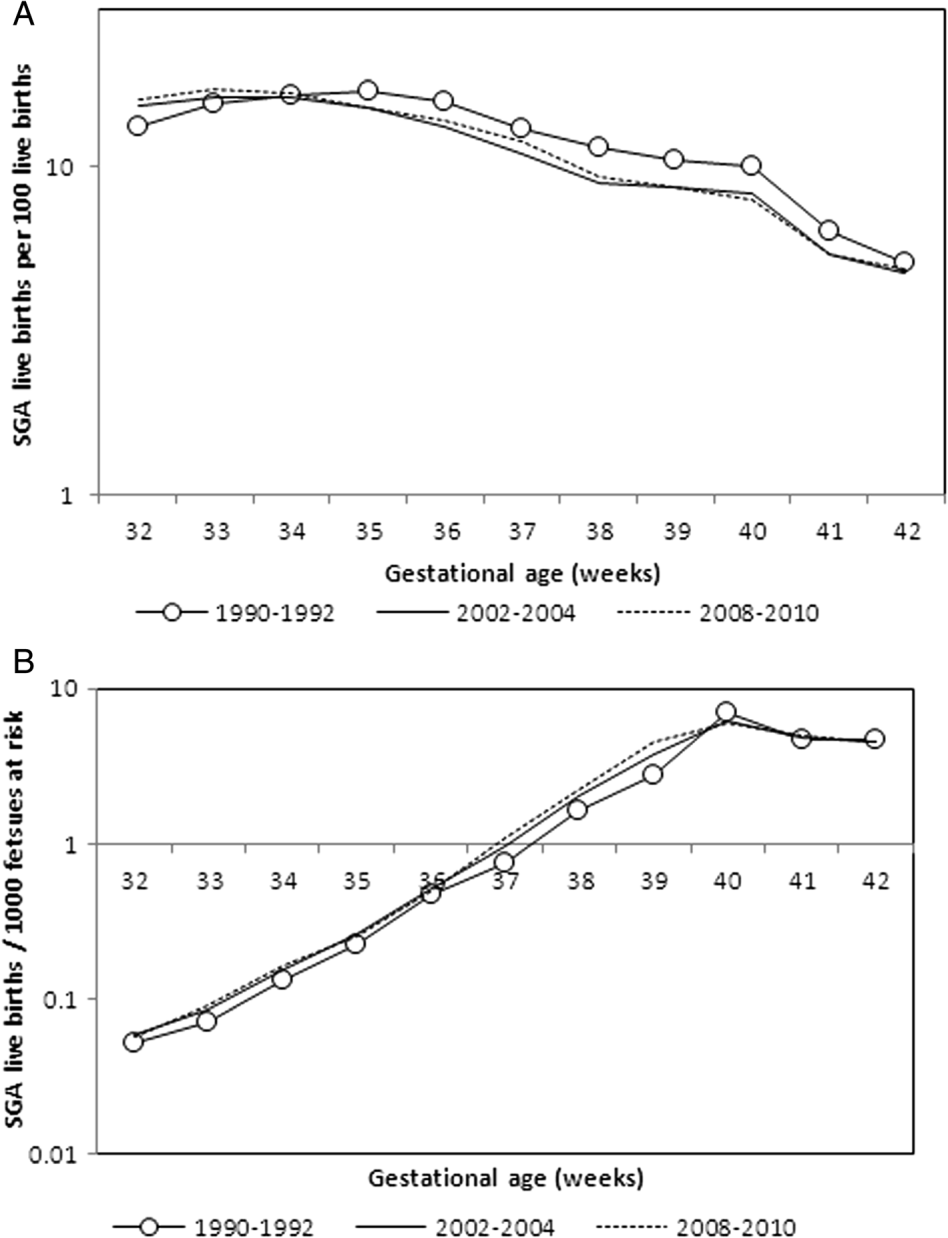
Changes in the incidence of birth (**a**) and small-for-gestational age (SGA) per 1000 fetuses at risk (**b**), singleton live births in the United States 1990–92, 2002–04, 2008–10

Figure 2 shows changes in the gestational age distribution (incidence of birth) in 1990–92, 2002–04 and 2008–10 and also changes in gestational age-specific SGA rates based on the fetuses-at-risk approach. Birth rates increased substantially between 1990–92 and 2002–04 (corresponding to the large increases in preterm and early term birth mentioned above). Between 2002–04 to 2008–10 the incidence of birth decreased at preterm gestation and increased at 39 weeks (corresponding to the small decline in preterm and early term birth mentioned above). SGA rates increased with increasing gestation and SGA incidence rates were higher in 2002–04 compared with 1990–92 at each gestational week between 32 and 39 weeks. In 2008–10, SGA rates at 37–39 weeks were slightly higher than SGA rates in 2002–04.

Table 1 shows the changes in maternal, obstetric and infant characteristics between 1990–92, 2002–04 and 2008–10 and rates of SGA within levels of each factor. SGA rates declined between 1990–92 and 2002–04 within categories of most risk factors and this was followed by a small increase or stabilization in SGA rates between 2002–04 and 2008–10 (Table 1). For instance, SGA rates among smokers declined from 17.6 % in 1990–92, to 15.7 % in 2002–04 and then increased slightly to 15.9 %, while among nulliparous women aged ≥35 years, SGA rates were 12.3 % in 1990–92, 10.8 % in 2002–04 and 11.1 % in 2008–10.

**Table 1:** Changes in population characteristics and small-for-gestational age (SGA) rates in the United States 1990–1992, 2002–2004, 2008–2010

Characteristic	1990–1992		2002–04		2008–10	
	All live births	SGA	All live births	SGA	All live births	SGA
	No. (%)	No. (rate/100)	No. (%)	No. (rate/100)	No. (%)	No. (rate/100)
Age <20 years	1,266,799 (13.1)	178,826 (14.1)	1,088,005 (10.8)	143,644 (13.2)	1,190,869 (10.1)	156,115 (13.1)
20–24 years	2,565,257 (26.6)	285,798 (11.1)	2,611,711 (25.9)	272,080 (10.4)	2,900,487 (24.6)	307,631 (10.6)
25–29 years	2,890,561 (29.9)	260,847 (9.0)	2,699,395 (26.8)	214,646 (8.0)	3,346,378 (28.4)	279,361 (8.3)
30–34 years	2,069,631 (21.4)	174,713 (8.4)	2,345,977 (23.3)	162,548 (6.9)	2,721,282 (23.1)	202,789 (7.5)
35–39 years	747,403 (7.7)	66,494 (8.9)	1,097,056 (10.9)	82,088 (7.5)	1,335,357 (11.3)	101,957 (7.6)
≥40 years	117,194 (1.2)	11,932 (10.2)	239,552 (2.4)	21,858 (9.1)	311,398 (2.6)	27,668 (8.9)
Non-Hispanic White	6,636,243 (68.7)	540,896 (8.2)	6,132,920 (60.8)	438,101 (7.1)	6,359,020 (53.9)	471,413 (7.4)
Non-Hispanic Black	1,700,294 (17.6)	291,888 (17.2)	1,559,920 (15.5)	231,876 (14.9)	1,731,740 (14.7)	262,800 (15.2)
Hispanic	992,365 (10.3)	106,957 (10.8)	1,857,538 (18.4)	167,041 (9.0)	2,901,453 (24.6)	248,242 (8.6)
Other	327,943 (3.4)	38,869 (11.9)	531,318 (5.3)	59,846 (11.3)	813,558 (6.9)	93,066 (11.4)
Low education	2,007,942 (21.7)	289,581 (14.4)	2,061,474 (20.7)	246,226 (11.9)	2,340,804 (20.1)	266,128 (11.4)
Mother not married	2,756,978 (28.6)	416,329 (15.1)	3,548,727 (35.2)	434,968 (12.3)	4,850,493 (41.1)	568,102 (11.7)
Maternal smoking	1,504,155 (17.7)	264,893 (17.6)	1,112,652 (11.3)	174,247 (15.7)	984,727 (9.5)	156,269 (15.9)
Nulliparous	3,160,790 (32.9)	368,815 (11.7)	3,339,247 (33.3)	365,533 (10.9)	4,000,839 (34. 3)	450,705 (11.3)
Nulliparous, ≥35 years	118,162 (1.2)	14,550 (12.3)	209,556 (2.1)	22,694 (10.8)	266,465 (2.3)	29,673 (11.1)
Labour induced	1,097,842 (11.4)	99,493 (9.1)	2,315,760 (23.0)	199,300 (8.6)	2,800,695 (23.7)	263,567 (9.4)
Cesarean delivery	2,100,393 (21.8)	216,111 (10.3)	2,631,066 (26.1)	248,880 (9.5)	3,674,107 (31.1)	349,172 (9.5)
Infant sex (male)	4,942,269 (51.2)	397,264 (8.0)	5,160,195 (51.2)	359,953 (7.0)	6,042,020 (51.2)	427,464 (7.1)
Gest. age <32 weeks	106,575 (1.1)	7926 (7.4)	112,312 (1.1)	10,745 (9.6)	125,037 (1.1)	12,083 (9.7)
32–33 weeks	82,200 (0.9)	11,889 (14.5)	91,770 (0.9)	14,557 (15.9)	102,715 (0.9)	17,192 (16.7)
34–36 weeks	478,843 (5.0)	77,605 (16.2)	637,780 (6.3)	90,439 (14.2)	721,151 (6.1)	105,742 (14.7)
37–38 weeks	1,721,215 (17.8)	206,315 (12.0)	2,763,381 (27.4)	262,380 (9.5)	3,339,299 (28.3)	337,035 (10.1)
39–40 weeks	5,712,036 (59.2)	580,887 (10.2)	5,581,621 (55.4)	470,764 (8.4)	6,698,762 (56.7)	559,528 (8.4)
41 weeks	1,160,789 (12.0)	73,403 (6.3)	805,794 (8.0)	43,635 (5.4)	760,526 (6.4)	40,911 (5.4)
≥42 weeks	395,187 (4.1)	20,585 (5.2)	89,038 (0.9)	4344 (4.9)	58,281 (0.5)	3030 (5.2)
Total	9,656,845 (100)	978,610 (10.1)	10,081,696 (100)	896,864 (8.9)	11,805,771 (100)	1,075,521 (9.1)

Low education denotes less than high school education. Gestational age-specific SGA rates were calculated using the traditional approach (per 100 live births in that gestational age category)

Table 2 presents the absolute and relative differences in SGA by gestational age group. The decline in overall SGA rates between 2002–04 and 2008–10 was associated with declines in SGA rates among infants born between 39–40 weeks and ≥42 weeks. The small increase in SGA rates between 2002–04 and 2008–10 was associated with small but significant increases in SGA rates among infants at 37–38, 39–40, 41 and ≥42 weeks (Table 2).

**Table 2:** Small-for-gestational age (SGA) rates, rate ratios and rate differences by gestational age among singleton live births in the United States (1990–1992 vs. 2002–2004; 2002–2004 vs. 2008–2010)

Gestational Age (weeks)	Fetuses-at-Risk (FAR) Approach			
	1990–1992	2002–2004	2002–2004 vs. 1990–1992	
	SGA per 100 FAR	SGA per 100 FAR	Rate Difference	Rate Ratio (95 % CI)
24–33 weeks	0.21	0.25	+0.05	1.22 (1.20–1.25)
34–36 weeks	0.82	0.92	+0.10	1.12 (1.11–1.13)
37–38 weeks	2.30	2.84	+0.54	1.24 (1.23–1.24)
39–40 weeks	7.99	7.27	−0.72	0.91 (0.91–0.91)
41 weeks	4.72	4.88	+0.16	1.03 (1.02–1.05)
≥42 weeks	5.21	4.88	−0.33	0.94 (0.91–0.97)
Total	10.1	8.89	−1.23	0.88 (0.88–0.88)
ᅟ	ᅟ	ᅟ	ᅟ	ᅟ
	2002–2004	2008–2010	2008–2010 vs. 2002–2004	
	SGA per 100 FAR	SGA per 100 FAR	Rate Difference	Rate Ratio (95 % CI)
24–33 weeks	0.25	0.25	0.0	0.99 (0.97–1.00)
34–36 weeks	0.92	0.91	−0.01	1.00 (0.99–1.01)
37–38 weeks	2.84	3.10	+0.26	1.09 (1.09–1.10)
39–40 weeks	7.27	7.44	+0.17	1.02 (1.02–1.03)
41 weeks	4.88	5.00	+0.12	1.02 (1.01–1.04)
≥42 weeks	4.88	5.20	+0.32	1.07 (1.01–1.12)
Total	8.89	9.11	+0.21	1.02 (1.02–1.03)

The modified Kitagawa decomposition showed that the initial decline in SGA rates was entirely due to changes in the gestational age distribution (Table 3). Furthermore, the decomposition showed that changes in gestational age-specific rates between 1990–92 and 2002–04 acted to increase SGA rates (Table 3); the largest change occurred among infants born at term gestation. In the later time period (2002–04 to 2008–10) as well, changes in the gestational age distribution and changes in gestational-age-specific SGA rates acted in opposite directions. The latter changes, which acted to increase SGA rates, overwhelmed the effect of changes in the gestational age distribution (Table 3).

**Table 3:** Relative contribution of changes in the gestational age distribution and in gestational age-specific small-for-gestational age (SGA) rates to the overall temporal changes in sga rates among singleton live births in the United States (1990–1992 vs. 2002–2004; 2002–2004 vs. 2008–2010)

Period	Gestational Age (weeks)	Modified Kitagawa decomposition (per 10,000 fetuses-at-risk)				
		Contribution of changes in		Total change	Relative contribution of changes in	
		Gestational age	Gestational age-specific SGA		Gestational age (%)	Gestational age-specific SGA (%)
1990–1992 vs. 2002–2004	24–33 weeks	0.00	4.58	4.58	0.0	100.0
	34–36 weeks	−0.37	9.71	9.34	−4.0	104.0
	37–38 weeks	−8.83	55.44	46.61	−18.9	118.9
	39–40 weeks	−168.20	33.63	−134.57	125.0	−25.0
	41 weeks	−34.71	1.98	−32.73	106.0	−6.0
	≥42 weeks	−16.53	−0.48	−17.01	97.2	2.8
	Total	−228.65	104.87	−123.78	184.7	−84.7
2002–2004 vs. 2008-2010	24–33 weeks	0.01	−0.31	−0.30	−3.3	103.3
	34–36 weeks	0.12	−0.26	−0.14	−85.7	185.7
	37–38 weeks	0.48	24.75	25.23	1.9	98.1
	39–40 weeks	−38.16	45.14	6.99	−546.2	646.2
	41 weeks	−9.58	0.95	−8.63	111.0	−11.0
	≥42 weeks	−1.84	0.09	−1.74	105.3	−5.3
	Total	−48.96	70.36	21.41	−228.7	328.6

Similar to the findings among all singletons, the SGA rate among smokers declined from 1990–92 to 2002–04 and increased slightly in 2008–10 (Table 1). Rates of induction and/or caesarean delivery among smokers increased from 30.2 % in 1990–92 to 46.2 % in 2002–04 and to 53.6 % in 2008–10. Temporal changes in the gestational age distribution and in gestational-age-specific SGA rates were similar among smokers and among all singletons. The overall temporal trends in SGA and the overall Kitagawa attribution of declines in SGA rates to changes in the gestational age distribution and to changes in gestational age-specific SGA rates were similar among smokers and among all singletons (Appendix Table 4 and 5). Overall declines in SGA rates among smokers were associated with declines in SGA rates at 39–40 weeks and at ≥42 weeks (although the latter change was not significant). Similarly, the results of the modified Kitagawa decomposition among smokers were similar to the findings among all singletons (Appendix Table 5); the large decline in SGA rates among smokers between 1990–92 and 2002–04 was due to changes in the gestational age distribution, while the small increase in SGA rates among smokers between 2002–04 and 2008–10 was due to changes in the gestational age-specific rates of SGA.

## Discussion

Temporal changes in SGA rates among singletons in the United States followed a non-linear pattern, with substantial declines between 1990–92 and 2002–04 and a small increase between 2002–04 and 2008–10. These declines in SGA rates were not consistent with the observed pattern of changes in the frequency of risk factors for fetal growth restriction such as maternal smoking, which decreased monotonically from 1990–92 to 2008–10. The SGA pattern was also not consistent with changes in older maternal age and nulliparity, both of which increased in a linear fashion between 1990–92 and 2008–10. The complex pattern of changes in SGA rates was more consistent with changes in the gestational age distribution; rates of late preterm birth, and early term birth increased substantially between 1990–92 and 2002–04 before declining in the period between 2002–04 and 2008–10. In our study, the sensitivity analyses restricted to smokers showed similar overall patterns in obstetrical intervention and SGA indicating that changing maternal characteristics (such as declines in maternal smoking) cannot explain temporal trends in SGA rates. Kitagawa decomposition analyses using the fetuses-at-risk approach supported these findings by showing that the decline in the SGA rate between 1990–92 and 2002–04 was entirely due to large changes in the gestational age distribution, whereas the small increase in SGA rates between 2002–04 and 2008–10 was due to adverse changes in gestational age-specific rates (which overwhelmed smaller positive changes in the gestational age distribution). Increases in gestational age-specific rates of SGA between 2002–04 and 2008–10 adversely impacted overall SGA rates-presumably the adverse effects of increases in older maternal age, and nulliparity on fetal growth were stronger than those of declines in maternal smoking and increases in pre-pregnancy weight.

There is some uncertainty in the literature with regard to the utility of the fetuses-at-risk approach for modeling postnatal phenomena such as SGA live births [16–19]. The traditional model, which shows stable SGA rates across pregnancy, receives some support from observed increases in birthweight with increasing gestational age that result from increases in uterine artery blood flow and other physiologic changes that occur through the course of pregnancy. Changes in fetal growth over the last two decades under this model are best viewed as the consequence of increases in some risk/protective factors (e.g., nulliparity, older maternal age, and pre-pregnancy obesity) and decreases in others (e.g., maternal smoking). However, the constancy of growth restriction across gestation is difficult to reconcile with changes in fetal and perinatal mortality with increasing gestation.

The fetuses-at-risk model, on the other hand, shows that rates of SGA increase with advancing gestation. The utero-placental system’s ability to support the fetus declines over the course of pregnancy and this viewpoint is supported by numerous animal and human studies which show that blood flow per unit fetal weight declines toward later gestation [20–23]. Early delivery, whether at late preterm, early term or late term gestation, will result in lower rates of SGA. This does not necessarily imply improved neonatal health status; indeed the DIGITAT trial which showed a reduction in SGA (<3rd percentile) from 30.6 % in the expectant management group to 12.5 % in the labour induction group did not show a significant difference in neonatal mortality or morbidity rates [5].

The reasons for the observed increase in obstetrical intervention are poorly understood. A proportion of this increase can be explained by changing maternal characteristics such as increased rates of delayed childbearing and obesity which increase pregnancy complications such as hypertension and diabetes [24]. Additionally, with technological advances in fetal surveillance and monitoring, clinicians are now more aware of subtle changes in fetal wellbeing [2]. However, in general clinical practice it is estimated that less than one-third of fetal growth restriction is identified antenatally [25]. It is estimated that much of the increase in obstetrical intervention for term deliveries appears to have been for informal medical indications or non-medical reasons [8, 26, 27]. Also, a lower threshold for intervention in recent times may relate to the safety of labour induction and the relative rarity of severe neonatal morbidity and mortality after 34 weeks of gestation [2]. None of this contradicts the proposition that SGA rates, which increase with advancing gestation under the fetuses-at-risk model, will decline with a left-shift in the gestational age distribution. In recent years, clinical guidelines have recommended that elective deliveries be scheduled in the late-term period (i.e. 39 weeks) [28]. While this has likely contributed to changes in the gestational age distribution with reductions in late preterm/early term births, both randomized controlled trials and observational studies of routine clinical practice have not found a significant impact on short-term maternal or neonatal health outcomes [29, 30]. The impact of these policies on long-term neurological development is currently unknown.

This study has both strengths and limitations. Its large size (*n* = 71,678,794 live births) provided the power to examine gestational age-specific phenomena. Also the use of the Kitagawa decomposition provided insight into temporal changes in SGA rates. We chose this method of analysis instead of logistic regression because of difficulty in separating the risk factors for SGA from the indications and effects of obstetric intervention. For instance, labour induction can prevent SGA in a fetus that is falling off its normal growth trajectory but is also used to deliver SGA fetuses before further deterioration in health status. An inability to distinguish the protective effect of labour induction because of confounding by indication and the temporal ambiguity in the labour induction-SGA association compromises our ability to determine the role of obstetrical intervention on temporal trends in SGA using regression methods. Reductions in SGA due to labour induction can be erroneously attributed to the indications and other correlates of induction (such as hypertension, diabetes, older maternal age and smoking).

Limitations of our study include use of data from US birth certificates which may contain some transcription and other errors. Several studies have confirmed that the accuracy of this data varies by specific data element [31–33]. Additionally, US birth certificates do not collect the indication for labour induction or cesarean delivery [34] and validation studies show that birth certificates over-estimate the rate of obstetrical intervention [34]. While misclassification is a concern, no studies have indicated that this misclassification is differential over time [32]. The increased use of ultrasound for pregnancy dating may have impacted gestational age trends in our study [35–38]. Ultrasonography for pregnancy dating (which would be reflected in the clinical estimate of gestational age) leads to an artefactual shift in the gestational age distribution with fewer post-term births compared to menstrual-based estimates of gestational age. Other limitations included our inability to distinguish elective cesarean delivery (which shortens gestational duration) from emergency cesarean delivery (which does not materially alter gestational duration). Also, our data source did not include information on pre-pregnancy weight; increases in pre-pregnancy obesity are another potential explanation for the temporal changes in SGA rates. Information on other interventions such as the use of assisted reproductive technologies which have been shown to result in shorter lengths of gestation and lower birth weights than naturally conceived pregnancies [39–41], was not consistently available throughout the study period and hence not examined as a potential explanatory factor for the temporal changes in SGA rates. Our study assumed that increases in early delivery rates were due to increases in obstetric intervention. However, some increase in spontaneous preterm birth and spontaneous early term birth may have occurred because of increases in other factors such as multiple births, older maternal age and parity. Studies which have documented a left shift in the gestational age distribution for spontaneous deliveries show a shift from delivery at ≥40 weeks to delivery at 39 weeks of gestation [42, 43]. However, these studies also show that the rate of scheduled births has increased, and the gestational age at which obstetric interventions are used has decreased [43]. Finally, we used neonatal growth curves instead of fetal growth curves, although the former are known to underestimate SGA at preterm gestation. We chose to use neonatal curves because our study was based on SGA as assessed among live births; fetal growth curves provide ultrasound-based fetal weight and this corresponds poorly with birth weight, especially at the extremes.

## Conclusions

In conclusion, our study showed that complex patterns in SGA rates between 1990–92 and 2008–10 cannot be explained by the linear pattern of changes in factors such as maternal smoking. The Kitagawa decomposition method based on the fetuses-at-risk approach also showed that declines in SGA rates between 1990–92 and 2002–04 occurred primarily due to changes in the gestational age distribution, while the small increase in SGA rates between 2002–04 and 2008–2010 was the result of changes in gestational age-specific SGA rates overwhelming smaller changes in the gestational age distribution. Our study provides a caveat for causal studies using non-experimental methods in situations where the natural course of disease phenomena is affected by clinical actions designed to alter outcomes. Control for time-varying confounding associated with changing obstetrical practice patterns may be necessary to elucidate true exposure-outcome relationships.

## Appendix

**Table 4:** Small-for-gestational age (SGA) rates, rate ratios and rate differences by gestational age among singleton live births to smokers in the United States (1990–1992 vs. 2002–2004; 2002–2004 vs. 2008–2010)

Gestational Age (weeks)	Fetuses-at-Risk (FAR) Approach			
	1990–1992	2002–2004	2002–2004 vs. 1990–1992	
	SGA per 100 FAR	SGA per 100 FAR	Rate Difference	Rate Ratio(95 % CI)
24–33 weeks	0.3	0.3	+0.06	1.20 (1.15–1.26)
34–36 weeks	1.4	1.6	+0.22	1.16 (1.14–1.18)
37–38 weeks	4.2	5.5	+1.28	1.30 (1.29–1.32)
39–40 weeks	14.3	13.2	−1.14	0.92 (0.91–0.93)
41 weeks	9.0	9.0	−0.01	1.00 (0.97–1.02)
≥42 weeks	9.9	9.5	−0.38	0.96 (0.90–1.03)
Total	17.6	15.7	−1.95	0.89 (0.88–0.89)
ᅟ	ᅟ	ᅟ	ᅟ	ᅟ
	2002–2004	2008–2010	2008–2010 vs. 2002–2004	
	SGA per 100 FAR	SGA per 100 FAR	Rate Difference	Rate Ratio (95 % CI)
24–33 weeks	0.3	0.4	+0.01	1.04 (0.99–1.09)
34–36 weeks	1.6	1.6	−0.01	0.99 (0.97–1.02)
37–38 weeks	5.5	6.0	+0.45	1.08 (1.07–1.10)
39–40 weeks	13.2	13.4	+0.20	1.02 (1.01–1.03)
41 weeks	9.0	9.2	+0.20	1.02 (0.99–1.06)
≥42 weeks	9.5	10.0	+0.55	1.06 (0.94–1.18)
Total	15.7	15.9	+0.21	1.01 (1.01–1.02)

**Table 5:** Relative contribution of changes in the gestational age distribution and in gestational age-specific small-for-gestational age (SGA) rates to temporal changes in sga rates among singleton live births to smokers in the United States (1990–1992 vs. 2002–2004; 2002–2004 vs. 2008–2010)

Period	Gestational Age (weeks)	Modified Kitagawa decomposition (per 10,000 fetuses-at-risk)				
		Contribution of changes in		Total change	Relative contribution of changes in	
		Gestational age	Gestational age-specific SGA		Gestational age (%)	Gestational age-specific SGA (%)
1990–1992 vs. 2002–2004	24–33 weeks	0.00	5.71	5.71	−0.1	100.1
	34–36 weeks	−0.90	22.55	21.66	−4.1	104.1
	37–38 weeks	−20.04	126.93	106.89	−18.8	118.8
	39–40 weeks	−300.73	65.70	−235.02	128.0	−28.0
	41 weeks	−61.97	−0.14	−62.11	99.8	0.2
	≥42 weeks	−31.95	−0.20	−32.15	99.4	0.6
	Total	−415.59	220.57	−195.02	213.1	−113.1
2002–2004 vs. 2008–2010	24–33 weeks	0.02	1.22	1.24	1.6	98.4
	34–36 weeks	0.22	−1.07	−0.86	−25.3	125.3
	37–38 weeks	0.25	41.99	42.25	0.6	99.4
	39–40 weeks	−77.13	77.82	0.68	−11290.7	11390.7
	41 weeks	−20.00	1.46	−18.54	107.9	−7.9
	≥42 weeks	−4.01	0.12	−3.89	103.2	−3.2
	Total	−100.67	121.54	20.88	−482.2	582.2

## Abbreviations

DIGITAT: Disproportionate Intrauterine Growth Intervention Trial at Term
NCHS: National Centre for Health Statistics
SGA: Small for gestational age
US: United States
